# Risk of Contrast-Associated Acute Kidney Injury in Patients Undergoing Peripheral Angiography with Carbon Dioxide Compared to Iodine-Containing Contrast Agents: A Systematic Review and Meta-Analysis

**DOI:** 10.3390/jcm11237203

**Published:** 2022-12-04

**Authors:** Gernot Wagner, Anna Glechner, Emma Persad, Irma Klerings, Gerald Gartlehner, Deddo Moertl, Sabine Steiner

**Affiliations:** 1 Department for Evidence-Based Medicine and Evaluation, University for Continuing Education Krems, Dr.-Karl-Dorrek-Strasse 30, 3500 Krems, Austria; 2 RTI International, 3040 East Cornwallis Road, Research Triangle Park, NC 27709-2194, USA; 3 Karl Landsteiner University of Health Sciences, Dr.-Karl-Dorrek-Strasse 30, 3500 Krems, Austria; 4 Department of Internal Medicine, University Hospital St. Poelten, Dunant-Platz 1, 3100 St. Poelten, Austria; 5 Department of Internal Medicine, Neurology and Dermatology, Division of Angiology, University Hospital Leipzig, Liebigstrasse 20, 04103 Leipzig, Germany; 6 Helmholtz Institute for Metabolic, Obesity and Vascular Research (HI-MAG) of the Helmholtz Zentrum Muenchen, University Hospital Leipzig and University of Leipzig, Liebigstrasse 20, 04103 Leipzig, Germany

**Keywords:** contrast-associated acute kidney injury, peripheral angiography, peripheral vascular intervention, carbon dioxide, systematic review

## Abstract

The aim of this systematic review was to summarise the comparative evidence on the risk of contrast-associated acute kidney injury (CA-AKI) with CO_2_ or iodinated contrast medium (ICM) for peripheral vascular interventions. We searched Ovid MEDLINE, Cochrane Library, Embase, Epistemonikos, PubMed-similar-articles, clinical trial registries, journal websites, and reference lists up to February 2022. We included studies comparing the risk of CA-AKI in patients who received CO_2_ or ICM for peripheral angiography with or without endovascular intervention. Two reviewers screened the references and assessed the risk of bias of the included studies. We extracted data on study population, interventions and outcomes. For the risk of CA-AKI as our primary outcome of interest, we calculated risk ratios (RRs) with a 95% confidence interval (CI) and performed random-effects meta-analyses. We identified three RCTs and five cohort studies that fully met our eligibility criteria. Based on a random-effects meta-analysis, the risk of CA-AKI was lower with CO_2_ compared to ICM (8.6% vs. 15.2%; RR, 0.59; 95% CI 0.33–1.04). Only limited results from a few studies were available on procedure and fluoroscopy time, radiation dose and CO_2_-related adverse events. The evidence suggests that the use of CO_2_ for peripheral vascular interventions reduces the risk of CA-AKI compared to ICM. However, due to the relevant residual risk of CA-AKI with the use of CO_2_, other AKI risk factors must be considered in patients undergoing peripheral vascular interventions.

## 1. Introduction

Peripheral vascular interventions (PVIs) are increasingly performed in patients with peripheral arterial disease (PAD) [1] and, in parallel, the complexity of the procedures is on the rise, as more patients with advanced age and multiple co-morbidities are treated.

During PVIs, the administration of an iodinated contrast medium (ICM) has been described as a risk factor for both acute kidney injury (AKI) and subsequent clinically relevant major adverse kidney events, such as persistently impaired renal function, need for new haemodialysis and death [2]. Although contrast-associated AKI (CA-AKI) is usually mild and creatinine levels typically return to baseline within two weeks [3,4,5], it is associated with an increased risk of serious adverse in-hospital and long-term outcomes, including all-cause mortality [6,7]. While the underlying pathophysiology of CA-AKI has not been fully elucidated, the proposed mechanisms include the direct cytotoxic effects of ICM on tubular cells as well as perturbed renal haemodynamics [5].

The reported risk of CA-AKI varies between 7% and 11% [2,4,8,9,10,11]. Substantially higher and lower rates were attributed to the use of different AKI definitions, different ICM administration modes (intravenous vs. intraarterial), ICM choice and dose, as well as the considerable heterogeneity in patient populations with respect to co-morbidities and the severity of underlying renal disease [2]. Pre-existing individual risk factors, particularly chronic kidney disease and the patient’s hydration status, are considered major risk determinants for the development of CA-AKI [10,12]. In previous randomised controlled trials (RCTs), no preventive measures, including hydration, sodium bicarbonate and acetylcysteine, could convincingly demonstrate a reduction in CA-AKI [13,14].

Importantly, it cannot be excluded that other factors beyond ICM contribute to the observed renal impairment and some researchers have even questioned whether ICM plays a significant role at all for the observed deterioration in kidney function [8,15], claiming that the risk of CA-AKI for the patient is overstated in the literature and overestimated by physicians [8,16]. Such overestimation of CA-AKI risk could even be harmful to patients if needed imaging procedures are delayed for unfounded fear of CA-AKI. Thus, it is of great scientific interest and clinical relevance to study the potential role of contrast-saving or contrast-avoiding strategies for the reduction of CA-AKI.

For PVI, it is possible to reduce the quantity of conventional ICM or even completely avoid it using carbon dioxide (CO_2_) as an alternative contrast agent [12]. CO_2_ acting as a negative contrast agent has been used for a variety of vascular procedures since the introduction of digital subtraction angiography. Because of its high solubility rate and rapid diffusibility via the lungs, CO_2_ is safe for peripheral intravascular use, but should not be used above the diaphragm to avoid the possibility of causing a cerebral air embolism, associated with stroke or death [17].

Current research on the clinical benefits and risks of CO_2_ angiography is limited and based on observational data and small RCTs. Therefore, we conducted a systematic review to summarise the comparative evidence on the risk of CA-AKI with CO_2_ or conventional ICM for peripheral angiography with or without endovascular intervention.

## 2. Materials and Methods

We followed the Preferred Reporting Items for Systematic Reviews and Meta-Analyses (PRISMA) guidelines in reporting the results of this systematic review [18].

### 2.1. Literature Search

An experienced information specialist (I.K.) conducted the following database searches from inception to February 2022: Ovid MEDLINE (accessed on 14 February 2022), Cochrane Library (Wiley) (accessed on 15 February 2022) and Embase.com (Elsevier, Netherlands) (accessed on 15 February 2022). In addition, we searched the following trial registries to identify unpublished and ongoing studies: ClinicalTrials.gov and the World Health Organization International Clinical Trials Registry Platform (ICTRP) (both accessed on 15 February 2022). Two sources were searched from inception to May 2021 but not updated because they had not identified any relevant studies: Epistemonikos.org (accessed on 19 May 2021) and PubMed-similar-articles (publications identified as potentially relevant in the preliminary search served as source references) (accessed on 18 May 2021). When possible, we combined controlled vocabulary (e.g., Medical Subject Headings (MeSH)) and free-text terms in the search strategies. We provide our full search strategies as Supplementary Table S1. In addition, we checked the reference lists of the included studies, published reviews and trial registry entries as well as the websites of journals in the vascular medicine field not indexed in the searched databases (e.g., Journal of Critical Limb Ischemia).

### 2.2. Eligibility Criteria and Study Selection

We included studies that compared the risk of CA-AKI injury in patients who received CO_2_ or ICM for angiography of the lower limb arteries, kidney arteries or infrarenal aorta with or without endovascular intervention (angioplasty, stent, endograft). We determined CA-AKI as our primary outcome of interest and included only studies that defined and reported CA-AKI as a binary outcome. Thus, studies reporting only continuous outcomes such as changes in creatinine or in the glomerular filtration rate (GFR) were excluded. For consistency, we will use the more current term CA-AKI throughout this manuscript instead of contrast-induced nephropathy, which was often used in the included studies. Studies that include patients receiving haemodialysis prior to the intervention were excluded. Appendix Table A1 shows the inclusion and exclusion criteria applied during literature screening in detail.

All references identified by our literature search were organised with Endnote X9.3 (Clarivate, PA, USA). We used the Covidence (Veritas Health Innovation, Melbourne, Australia) [19] online systematic review tool to screen references against our eligibility criteria. Two reviewers (G.W., A.G.) independently screened the references in two subsequent steps. First, they screened the references yielded by the systematic search based on title and abstract. Second, for those references considered relevant by both reviewers, full-text articles were retrieved and screened to ascertain whether the study met the eligibility criteria. At each step, conflicts were discussed and resolved between the two reviewers. If necessary, a third reviewer with extensive clinical expertise in the interventional angiology field (S.S.) was involved.

### 2.3. Risk of Bias and Certainty of Evidence

To assess the risk of bias, we used the Cochrane Risk of Bias 2 (RoB 2) tool [20] for RCTs and the Risk Of Bias In Non-Randomized Studies of Interventions (ROBINS-I) tool [21] for non-randomised studies. Different pairs of reviewers (A.G., E.P., G.W.) independently rated the risk of bias. Consensus was obtained through discussion. We employed the Grading of Recommendations Assessment, Development and Evaluation (GRADE) [22] approach to assess the certainty of evidence for our primary outcome of interest. We used the GRADEpro (McMaster University and Evidence Prime, Hamilton, ON, Canada) [23] online tool to create a GRADE evidence profile and summary of findings table.

### 2.4. Data Collection

We used electronic extraction tables to collect the following data items from the included studies: first author, year, country, study design, follow-up duration, recruitment period, inclusion and exclusion criteria, patient characteristics, intervention description including amount of CO_2_ and ICM, type of ICM, procedure and fluoroscopy time, radiation dose-area product, definition and risk of CA-AKI, additional adverse events and CO_2_-related side effects, and procedural outcome. One reviewer (G.W.) extracted the data into tables that a second reviewer (E.P.) checked for completeness and correctness.

### 2.5. Data Analysis

We conducted meta-analyses if the clinical heterogeneity among studies was reasonable. Based on the number of patients with CA-AKI and the number of patients at risk in each group, we calculated risk ratios (RRs) with 95% confidence intervals (CIs). We performed random-effects meta-analyses using the Paule–Mandel estimator of tau² [24]. To assess the statistical heterogeneity across studies, we visually inspected the forest plots and calculated the I^2^ statistics [25]. For CA-AKI, our primary outcome of interest, the number of events, patients at risk and effect estimates with 95% CI were presented as forest plots. The results of other outcomes were summarised and presented in tables. We conducted a sensitivity analysis with (1) studies rated as low and moderate/some risk of bias, (2) studies predominantly including patients with impaired renal function and (3) studies including more than 40% of patients with diabetes at baseline. We conducted a subgroup analysis according to the study design. If we had identified more than 10 studies, we intended to assess publication bias using a visual assessment of the funnel plots. For all analyses, we used the meta package [26] in RStudio (RStudio, PBC, Boston, MA, USA) [27] within the R environment (R Foundation for Statistical Computing, Vienna, Austria) [28].

## 3. Results

### 3.1. Study Characteristics

**Figure 1:**
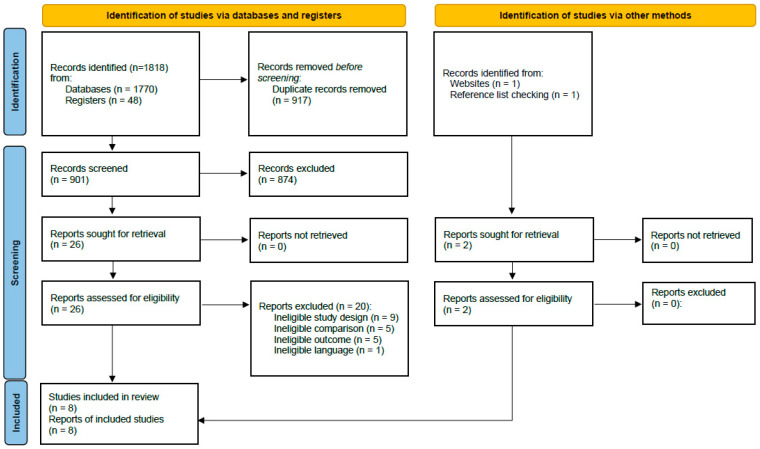
Preferred Reporting Items for Systematic Reviews and Meta-Analyses (PRISMA) flow-chart adapted from Page et al. 2021 [18].

After deduplication, our searches yielded 901 references. Of those, we included eight studies that fully met our eligibility criteria: three RCTs [29,30,31] and five cohort studies [32,33,34,35,36] (three retrospective and two prospective). The studies were published between the years 2001 and 2021. Participants were recruited between the years 1996 and 2018 and followed up for up to 6 months. The studies took place in Europe (Sweden, England, Germany), the United States, Egypt, Iran and Saudi Arabia. Figure 1 provides details of the study selection process. Notably, two of the included studies were published in journals not indexed in the searched databases. One study was identified through a trial registry entry and the other by hand searching the journal website. In Supplementary Table S2, we provide a list of references that were excluded based on a full-text assessment, including the reasons for exclusion.

### 3.2. Study Population

The included studies’ sample sizes ranged from 64 to 313 participants, and the reported mean age was between 54 and 78 years, with a majority being male. The proportion of participants with diabetes mellitus varied considerably in the individual studies, ranging from 17% to 65%. Most studies included participants with impaired renal function. Two studies only included patients with chronic kidney disease (CKD) stage 3 or higher (i.e., GFR < 60 mL/min) [33,34]. In four studies [31,32,35,36], participants’ serum creatinine at baseline was significantly higher in the CO_2_ group than in the ICM group.

Study participants received CO_2_ or ICM for angiography alone or combined with subsequent endovascular interventions. Most studies included patients with PAD as the underlying vascular condition [29,31,33,34,35]. One study each compared CO_2_ and ICM for endovascular infrarenal aortic aneurysm repair (EVAR) [32] or angiography of the renal artery with or without endovascular intervention [30]. One study included patients with diagnostic and therapeutic vascular procedures at different sites [36]. In patients with PAD, two studies reported a mean amount of 115 and 171 mL of CO_2_. Four publications did not provide this information. A small amount of ICM was applied in a variable number of patients in the CO_2_ groups in all studies. The control groups received ICM only.

The included studies’ authors used various definitions for CA-AKI. Commonly, an increase in serum creatinine of >25% or >0.5 mg/dL within 48 h, 72 h or even longer from baseline was considered CA-AKI. Notably, one study considered changes in creatinine up to 1 month. Table 1 and Table A2 summarise the characteristics of the included studies. Supplementary Table S3 presents the inclusion and exclusion criteria, recruitment periods and primary and secondary outcomes of each study.

### 3.3. Risk of Bias and Certainty of Evidence

For the RCTs, we assessed the risk of bias as low for one trial [29] and as some risk of bias for two trials [30,31]. We rated two cohort studies [33,34] as moderate and three [32,35,36] as serious risk of bias. Supplementary Figures S1 and S2 show the risk of bias ratings for each individual domain of the respective assessment tools. We graded the certainty of evidence as low for the RCTs’ findings and as very low for the cohort studies’. Supplementary Table S4 presents a detailed GRADE evidence assessment with a summary of findings.

### 3.4. Risk of CA-AKI

**Figure 2:**
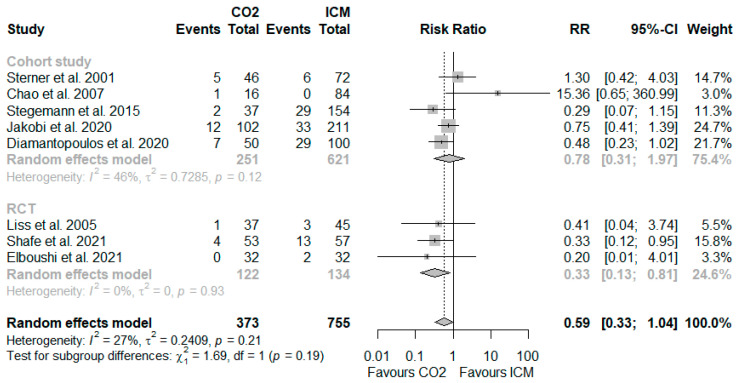
Forest plot for the risk of CA-AKI with CO_2_ compared to ICM [29,30,31,32,33,34,35,36]. Abbreviations: CA-AKI, contrast-associated acute kidney injury; CI, confidence interval; CO_2_, carbon dioxide; ICM, iodinated contrast medium; RCT, randomised controlled trial; RR, risk ratio.

Among the eight included studies with a total of 1128 participants, the risk of CA-AKI varied considerably and ranged up to 14% in the CO_2_ group and up to 29% in the ICM group (see Table 1). Based on a random-effects meta-analysis of eight studies, five rated as low or moderate/some risk of bias and three as serious risk of bias, the CA-AKI event rates were lower in participants receiving CO_2_ as a contrast agent compared to those exposed to ICM alone (8.6% vs. 15.2%; RR, 0.59; 95% CI 0.33–1.04; I^2^ = 27%; see Figure 2). The risk reduction was greater in a meta-analysis based on the RCTs (4.1% vs. 13.4%; RR, 0.33; 95% CI 0.13–0.81; I^2^ = 0%) as compared to those based on the cohort studies (10.8% vs. 15.6%; RR, 0.78; 95% CI 0.31–1.97; I^2^ = 46%). No patient required haemodialysis following the procedure in any of the studies reporting this outcome.

The largest study we identified was a recent single-centre prospective cohort study, including 313 participants with PAD and renal insufficiency (CKD stage 3 or higher) [34]. Overall, 102 participants who underwent PVI with CO_2_ were compared to 211 matched patients who received standard ICM [34]. The percentage of participants who experienced CA-AKI was lower in those who received less than 50 mL of ICM (6.8%) compared to those who received 51 to 100 mL (18.2%) or >100 mL (16.7%).

A sensitivity meta-analysis, including only low and moderate/some risk of bias studies, yielded a statistically significant lower risk of CA-AKI in participants receiving CO_2_ compared to those who received ICM (8.8% vs. 18.0%; RR, 0.55; 95% CI 0.36–0.83; I^2^ = 0%; see Supplementary Figure S3). A sensitivity analysis of the studies that predominantly included participants with impaired renal function (i.e., GFR < 60 mL/min) (RR, 0.69; 95% CI 0.45–1.06; I^2^ = 0%; see Supplementary Figure S4) or more than 40% patients with diabetes mellitus (RR, 0.52; 95% CI 0.35–0.79; I^2^ = 0%; see Supplementary Figure S5) rendered a lower risk of CA-AKI with CO_2_ than ICM. Supplementary Figures S6 and S7 show risk ratios in individual studies according to the type of intervention and amount of iodinated contrast medium administered in the control group.

### 3.5. Procedural Variables and Outcomes

In general, the technical success rate was 97% to 100% with CO_2_ and 95% to 100% with ICM [29,31,34,35]. Four studies reported on the procedure duration [29,32,34,35]. In the three studies comprising PAD patients undergoing PVI, the mean procedure duration with CO_2_ was in a similar range to that with ICM: 87 vs. 77 min [29], 83 vs. 79 min [35] and 92 vs. 102 min [34]. In a study of patients who received EVAR, the authors reported a significant difference of 3.0 vs. 2.3 h between the groups [32]. The radiation dose-area product differed substantially between studies and treatment arms [32,34,35].

### 3.6. Additional Adverse Events and CO_2_-Related Side Effects

The reporting of additional adverse events and CO_2_-related side effects differed considerably between studies. The number of vascular complications, however, was very low and the risk was similar in both groups. Four studies reported CO_2_-related side effects, such as nausea, vomiting and transient limb pain with variable frequency [30,33,34,35]. No deaths were reported during the follow-up of up to 6 months. Appendix Table A2 shows the procedural variables, additional adverse events and CO_2_-related side effects in detail.

## 4. Discussion

Based on RCTs and observational studies, this systematic review provides evidence that the use of CO_2_ for peripheral angiography with or without endovascular intervention reduces the risk of CA-AKI compared to conventional ICM. However, the certainty of evidence from RCTs is low because of imprecision due to the small number of events and small sample size of the included studies. The most common CO_2_-related adverse events included nausea, vomiting and transient leg pain. These were reported in only about half of the studies and the frequency varied widely between studies. Data on fluoroscopy time and radiation dose are also inconclusive because they were only reported in a few studies.

The findings of our review support the general concept of a renoprotective role of CO_2_ as an alternative contrast agent. Our results confirm the findings from a previous review and meta-analysis on the benefit of CO_2_ angiography, which involved substantially fewer patients [37]. In this review, the use of CO_2_ compared to ICM was also associated with a reduced risk of CA-AKI (4.3% vs. 11.1%, odds ratio (OR), 0.47; 95% CI 0.22–0.99) and this effect remained of the same magnitude in a relatively small subgroup analysis of four studies that included patients with CKD. However, the authors also included studies in which both the intervention and control groups received CO_2_ angiography with different doses of additional ICM. We used more stringent inclusion criteria comparing CO_2_ angiography with bailout ICM administration vs. ICM only, thus reflecting clinically relevant strategies.

The potential mechanistic pathway of the association between CA-AKI and adverse clinical events remains to be elucidated and it is still under dispute to what extent CA-AKI represents a mediator or risk marker, especially in mild AKI cases [38]. It cannot be excluded that other factors beyond ICM contribute to the observed renal impairment, and some researchers have even questioned whether ICM plays a significant role at all in the observed deterioration in kidney function [8,15]. Therefore, the term CA-AKI, rather than the previously used contrast-induced acute kidney injury, has been adopted. Importantly, CA-AKI occurred in over 8.8% of patients in the CO_2_ group in our meta-analysis, highlighting the role of additional harmful factors beyond the administration of ICM during vascular interventions.

Across the included studies, we observed a major difference regarding the risk of CA-AKI that could be explained by different causes. First, the different sites of peripheral angiographies (aortic, renal, infrainguinal) as diagnostic or interventional procedures required variable amounts of ICM. In addition, the complexity of the interventions has an impact on the amount of contrast agent. Second, the proportion of patients with diabetes mellitus and impaired renal function at baseline varied. Third, no uniform definition was used for CA-AKI. In particular, the time points and periods of post-procedural creatinine measurements were different. While our review supports a potential benefit of CO_2_ with respect to renal outcomes, we cannot draw conclusions about the frequency and severity of CO_2_-related side effects, as reporting was not sufficient in the included trials. Importantly, data on image quality were limited, which has been reported to be inadequate for clinical decision-making with CO_2_ angiography, especially for infrapopliteal interventions [39]. More research, ideally based on RCTs, is needed to describe the extent of renal protection and intraprocedural adverse events with CO_2_ angiography for vascular interventions.

This systematic review has the following limitations. First, we restricted our eligibility criteria to English and German publications. Second, publication bias could affect our findings since we could only rely on published data. Third, we observed heterogeneity regarding the baseline characteristics of the study participants, types of interventions and applied definition of CA-AKI.

Since most published data on the benefits of CO_2_ angiography focus on a small number of patients, further evidence from larger perspective RCTs is needed. There is also a lack of data regarding the association between ICM administration during PVI and clinically relevant composite outcomes, such as major adverse kidney events, consisting of persistently impaired renal function, need for new haemodialysis and death. The studies we identified rarely reported adverse events other than CA-AKI. If reported, the observation period was often very short.

In conclusion, the application of CO_2_ for PVI may reduce the risk of CA-AKI compared to ICM. However, a relevant residual risk of CA-AKI has been described with the use of CO_2_, indicating the influence of other risk factors. This should be considered when patients undergo PVI with CO_2_ for renal protection.

## Figures and Tables

**Table 1:** Key characteristics and outcome of the included studies.

Author, Year, Country	Study Design Risk of Bias Follow-Up	*N*	Age, Years, Mean (SD)	Women	Diabetes Mellitus	CKD Stage 3–5	Condition/Site and Intervention	CO_2_ and ICM Amount, ICM Type, (Mean ± SD or Median [IQR])	CA-AKI, Haemodialysis *n*/*N* (%) ^a^
Elboushi et al. 2021 [29] Saudi Arabia and Egypt	RCT Low 3 months ^b^	Total: 64 CO_2_: 32 ICM: 32	Age: CO_2_: 54.3 ± 9.8 ICM: 56.3 ± 9.7	Women: CO_2_: 29.0% ICM: 53.1%	Diabetes mellitus: All: 45.3% CO_2_: 55.0% ICM: 37.5%	CKD stage 3–5: CO_2_: 0 ICM: 0	PAD Angiography with aortoiliac endovascular intervention	CO_2_ amount, mL: CO_2_: 171 [NR] ICM: 0 ICM amount, mL: CO_2_: 10 [NR] (N = 3) ICM: 78 [NR] ICM type: Iohexol	CA-AKI: CO_2_: 0/32 ICM: 2/32 (6.3%) Haemodialysis: NR
Shafe et al. 2021 [31] Iran	RCT Some concerns 1 month	Total: 110 CO_2_: 53 ICM: 57	Age: CO_2_: 62.5 ± 8.4 ICM: 63.3 ± 11.7	Women: CO_2_: 24.5% ICM: 19.3%	Diabetes mellitus: All: 45% CO_2_: 51% ICM: 40%	CKD stage 3–5: CO_2_: NR ICM: NR	PAD Angiography without (29%) or with aortoiliac, femoropopliteal or infrapopliteal endovascular intervention (71%)	CO_2_ amount, mL: CO_2_: NR ICM: 0 ICM amount, mL: CO_2_: 11.4 ± 6.1 ICM: 93.2 ± 43.0 *ICM type:* NR	*CA-AKI:* CO_2_: 4/53 (7.5%) ICM: 13/57 (22.8%) Haemodialysis: CO_2_: 0/53 ICM: 0/57
Liss et al. 2005 [30] Sweden	RCT Some concerns 3 weeks	Total: 82 CO_2_: 37 ICM: 45	Age: CO_2_: 67 ± 8 ICM: 63 ± 11	Women: CO_2_: NR ICM: NR	Diabetes mellitus: All: 17% CO_2_: 27% ICM: 9%	CKD stage 3–5: CO_2_: NR ICM: NR	Renal arteries Angiography with or without endovascular intervention	CO_2_ amount, mL: CO_2_: 191 ± 118 ICM: 0 ICM amount, mL: CO_2_: 35.1 ± 6.4 ICM: 88.4 ± 42.9 *ICM type:* Ioxaglate	*CA-AKI:* CO_2_: 1/37 (2.7%) ICM: 3/45 (6.7%) Haemodialysis: CO_2_: 0/37 ICM: 1/45 (2.2%)
Sterner et al. 2001 [36] Sweden	Cohort study Serious 2 weeks	Total: 118 CO_2_: 46 ^c^ ICM: 72 ^d^	Age: CO_2_: 71 ± NR ICM: 72 ± NR	Women: CO_2_: 15% ICM: 33%	Diabetes mellitus: All: 21% CO_2_: 20% ICM: 22%	CKD stage 3–5: CO_2_: NR ICM: NR	PAD, Renal and mesenteric arteries ^d^ Angiography with or without endovascular intervention	CO_2_ amount, mL: CO_2_: NR ICM: 0 ICM amount, mL: CO_2_: 5 [NR] ICM: 22 [NR] ICM type: Iohexol	CA-AKI: CO_2_: 5/46 (10.9%) ICM: 6/72 (8.3%) Haemodialysis: NR
Chao et al. 2007 [32] USA	Cohort study Serious 6 months	Total: 100 CO_2_: 16 ICM: 84	Age: CO_2_: 77 ± NR ICM: 76 ± NR	Women: CO_2_: 6% ICM: 18%	Diabetes mellitus: All: 13% CO_2_: 20% ICM: 12%	CKD stage 3–5: CO_2_: 88% ICM: 34%	Abdominal aortic aneurysm EVAR	CO_2_ amount, mL: CO_2_: 50 ± NR ICM: 0 ICM amount, mL: CO_2_: 27 ± 5 ICM: 148 ± 20 ICM type: Iopamidol	CA-AKI: CO_2_: 1/16 (6.3%) ICM: 0/84 Haemodialysis: CO_2_: 0/16 ICM: 0/84
Stegemann et al. 2015 [35] Germany	Cohort study Serious NR	Total: 191 CO_2_: 37 ICM: 154	Age: CO_2_: 70 ± 10 ICM: 73 ± 12	Women: CO_2_: 38% ICM: 23%	Diabetes mellitus: All: 51% CO_2_: 51% ICM: 51%	CKD stage 3–5: CO_2_: 86% ICM: 29%	PAD Endovascular intervention (aortoiliac, femoropopliteal, below-the-knee)	CO_2_ amount, mL: CO_2_: NR ICM: 0 ICM amount, mL: CO_2_: 34 ± 41 ICM: 112 ± 76 ICM type: Iodixanol	*CA-AKI:* CO_2_: 2/37 (5%) ICM: 29/154 (19%) Haemodialysis: CO_2_: 0/37 ICM: 0/154
Diamantopoulus et al. 2020 [33] England	Cohort study Moderate 30 days	Total: 150 CO_2_: 50 ICM: 100	Age: CO_2_: 77.5 ± 10.4 ICM: 76.5 ± 10.5	Women: CO_2_: NR ICM: NR	Diabetes mellitus: All: 65% CO_2_: 66% ICM: 64%	CKD stage 3–5: CO_2_: 100% ICM: 100%	PAD Endovascular intervention (aortoiliac, femoropopliteal, below-the-knee)	CO_2_ amount, mL: CO_2_: NR ICM: 0 ICM amount, mL: CO_2_: 15.1 ± 14.0 ICM: 115.6 ± 58.1 ICM type: Iodixanol	CA-AKI: CO_2_: 7/50 (14%) ICM: 29/100 (29%) Haemodialysis: NR
Jakobi et al. 2021 [34] Germany	Cohort study Moderate 48 h	Total: 313 CO_2_: 102 ICM: 211	Age: CO_2_: 74.8 ± 8.7 ICM: 72.4 ± 9.3	Women: CO_2_: 36.3% ICM: 24.6%	Diabetes mellitus: All: 52% CO_2_: 51% ICM: 54%	CKD stage 3–5: CO_2_: 100% ICM: 100%	PAD Endovascular intervention (aortoiliac, femoropopliteal, below-the-knee)	CO_2_ amount, mL: CO_2_: 114.5 ± 53.4 ICM: 0 ICM amount, mL: CO_2_: 41.9 ± 31.6 (N = 86) ICM: 118.9 ± 51.1 ICM type: NR	CA-AKI: CO_2_: 12/102 (11.8%) ICM: 33/211 (15.6%) Haemodialysis: NR

Abbreviations: CA-AKI, contrast-associated acute kidney injury; CKD, chronic kidney disease; CO_2,_ carbon dioxide; EVAR, endovascular aortic aneurysm repair; ICM, iodinated contrast medium; IQR, interquartile range; NR, not reported; *n*, number of patients with events; *N*, number of patients at risk; mg, milligram; PAD, peripheral arterial disease; SD, standard deviation. ^a^: Percentages or number of events were self-calculated if not reported in the publication. ^b^: Based on the definition of outcomes. ^c^: Patients from group B (iodine + CO_2_) and group C (CO_2_) were added. ^d^: Patients in the control group underwent coronary (*n* = 25), pulmonary (*n* = 6), renal (*n* = 7), aortofemoral (*n* = 29), aortocervical (*n* = 4) and mesenteric. (*n* = 1) percutaneous endoluminal diagnosis and therapy procedures.

## Data Availability

All data analysed during this study are included in this published article and its Supplementary Materials.

## Supplementary Materials

The following supporting information can be downloaded at: https://www.mdpi.com/article/10.3390/jcm11237203/s1, Table S1: Search strategies; Table S2: Reasons for exclusion based on full-text assessment; Table S3. Inclusion and exclusion criteria as well as primary and secondary outcomes of included studies; Table S4. GRADE evidence profile and summary of findings for the comparison of CO_2_ and conventional ICM for peripheral angiography with or without vascular intervention; Figure S1: Risk-of-bias assessment for RCTs; Figure S2: Risk-of-bias assessment for cohort studies; Figure S3: Forest plot of the sensitivity analysis including only low and moderate/some risk of bias studies; Figure S4: Forest plot of the sensitivity analysis with studies predominantly including patients with impaired renal function (GFR < 60 mL/min); Figure S5: Forest plot of the sensitivity analysis with studies including more than 40% patients with diabetes; Figure S6: Forest plot with subgroups according to the type of intervention; Figure S7: Forest plot with subgroups according to the mean or median amount of iodinated contrast medium administered in the control group.

## Appendix A

**Table A1:** Study eligibility criteria.

	Included	Excluded
Populations	Adult patients undergoing angiography of the lower limb arteries, kidney arteries or infrarenal aorta with or without endovascular intervention	Children Angiography of other arteries (e.g., coronary angiography) Patients who required haemodialysis prior to the intervention
Intervention	Application of CO_2_ with or without supplemental use of a small amount of iodinated contrast medium	Any other intervention
Comparator	Application of conventional iodinated contrast medium only	Application of CO_2_ Any other comparator
Outcomes	Primary outcome: Risk of contrast-associated acute kidney injury as defined by study authors Secondary outcomes: Need for haemodialysis after the intervention Additional adverse events (e.g., vascular complications) CO_2_-related side effects (e.g., nausea, vomiting, limb pain) Procedural outcomes (e.g., technical success)	Post-procedural increase in creatinine/decrease in GFR without distinct definition or classification of contrast-associated acute kidney injury Other surrogate outcomes
Study designs	RCT Non-randomised controlled trials Controlled cohort studies	Cohort studies without control group Narrative and systematic reviews Case reports and case series
Publication type	Full publication	Abstracts only Letters and editorials
Publication language	English, German	All other languages

Abbreviations: CO_2,_ carbon dioxide; GFR, glomerular filtration rate; RCT, randomised controlled trial.

**Table A2:** Additional characteristics and outcome of the included studies.

Author, Year, Country	Pre-Intervention, GFR, Serum Creatinine (Mean ± SD or Median [IQR])	Procedure Time (Mean ± SD or Median [IQR])	Fluoroscopy Time, Radiation Dose-Area Product (Mean ± SD or Median [IQR])	Definition of Contrast-Induced Nephropathy	Additional Adverse Events, CO_2_-Related Side Effects and Procedural Outcome *n*/*N* (%) ^a^	
Elboushi et al. 2021 [29] Saudi Arabia and Egypt	GFR, mL/min: CO_2_: NR ICM: NR Creatinine, mg/dL: CO_2_: 0.92 ± 0.16 ICM: 0.94 ± 0.2	Procedure time, minutes: CO_2_: 87 ± 22 ICM: 77 ± 28	Fluoroscopy time: NR Radiation dose-area product: NR	Increase in serum creatinine exceeding 25% or more than or equal to 0.5 mg/dL within 1 month. ^b^	Technical success rate: CO_2_: 32/32 (100%) ICM: 32/32 (100%) No cardiac death, myocardial infarction, stroke and/or death within 3 months	Groin hematoma: CO_2_: 2/31^c^ (6.5%) ICM: 2/32 (6.3%) Pseudoaneurysm: CO_2_: 1/31 ^c^ (3.2%) ICM: 0/32 Major amputation: CO_2_: 2/31 ^c^ (6.5%) ICM: 3/32 (9.4%)
Shafe et al. 2021 [31] Iran	GFR, mL/min: CO_2_: 60.9 ± 22.0 ICM: 74.7 ± 23.6 Creatinine, mg/dL: CO_2_: 1.46 ± 0.45 ICM: 1.13 ± 0.28	Procedure time: NR	Fluoroscopy time: NR Radiation dose-area product: NR	Increase in serum creatinine exceeding 25% or 0.5 mg/dL within 72 h after the procedure	Technical success rate: CO_2_: 53/53 (100%) ICM: 57/57 (100%) Lower-limb pain: CO_2_: 12/53 (22.6%) ICM: 0/57	Major vascular complications: CO_2_: 0/53 ICM: 0/57 Death: CO_2_: 0/53 ICM: 0/57
Liss et al. 2005 [30] Sweden	GFR, mL/min: CO_2_: 54 ± 22 ICM: 59 ± 29 Creatinine, mg/dL: ^d^ CO_2_: 1.45 ± 0.43 ICM: 1.36 ± 0.42	Procedure time: NR	Fluoroscopy time: NR Radiation dose-area product: NR	Increase in serum creatinine by >25% within one week after the procedure	Vomiting: CO_2_: 1/37 (27.0%) ICM: 0/45	Nausea: CO_2_: 8/37 (21.6%) ICM: 1/45 (2.2%)
Sterner et al. 2001 [36] Sweden	GFR, mL/min: CO_2_: NR ICM: NR Creatinine, mg/dL ^e,f^ CO_2_: 2.57 [NR]; 3.71 [NR] ICM: 1.98 [NR]	Procedure time: NR	Fluoroscopy time: NR Radiation dose-area product: NR	Increase in serum creatinine by >25% within two weeks after the procedure		NR
Chao et al. 2007 [32] USA	GFR, mL/min: CO_2_: 36 ± NR ICM: 81 ± NR Creatinine, mg/dL: CO_2_: 1.8 ± NR ^g^ ICM: 1.0 ± NR	Procedure time, hours: CO_2_: 3.0 ± 0.3 ICM: 2.3 ± 0.2	Fluoroscopy time, minutes: CO_2_: 46 ± 7 ICM: 24 ± 1.5 Radiation dose-area product, cGy.cm^2^: CO_2_: 92,500 ± 13,800 ICM: 52,900 ± 4400	Increase in serum creatinine by >20% within 24 h after the procedure	Morbidity: CO_2_: 2/16 (12%) ICM: 5/84 (6%)	Death: CO_2_: 0/16 ICM: 0/84
Stegemann et al. 2015 [35] Germany	GFR, mL/min: CO_2_: 22 ± 34 ICM: 76 ± 28 Creatinine, mg/dL: CO_2_: 2.1 ± 1.3 ICM: 1.1 ± 0.6	Procedure time, minutes: CO_2_: 83 + 32 ICM: 79 + 37	Fluoroscopy time, minutes: CO_2_: 22 + 14 ICM: 23 + 17 Radiation dose-area product, cGy.cm^2^: CO_2_: 8054 + 12,764 ICM: 9359 + 11,474	Increase in serum creatinine by >25% or >0.5 mg/dL within 48 h after the procedure	Technical success rate: CO_2_: 37/37 (100%) ICM: 148/154 (96%)	Nausea: CO_2_: 1/37 (2.7%) ICM: 0/154 Several patients described temporary acute ischaemic lower leg pain following both ICM and CO_2_ injection.
Diamantopoulus et al. 2020 [33] England	GFR, mL/min: CO_2_: 38.6 ± 13.2 ICM: 43.3 ± 12.2 Creatinine, mg/dL:^f^ CO_2_: 1.7 ± 0.55 ICM: 1.54 ± 0.52	Procedure time: NR	Fluoroscopy time: NR Radiation dose-area product: NR	Increase in serum creatinine by >25% or >0.5 mg/dL within 72 h after the procedure	There were no major complications associated with the use of CO_2_. Most of the CLI cases reported transient discomfort (seconds) at the level of the symptomatic foot.	Major complications: CO_2_: 0/50 ICM: NR
Jakobi et al. 2021 [34] Germany	GFR, mL/min: CO_2_: 32.4 ± 11.8 ICM: 33.1 ± 15.6 Creatinine, mg/dL: CO_2_: NR ICM: NR	Procedure time, minutes: CO_2_: 92.3 ± 35 ICM: 101.8 ± 47.2	Fluoroscopy time: NR Radiation dose-area product, cGy.cm^2^: CO_2_: 6025 ± 6926 ICM: 4281 ± 4722	Increase in serum creatinine by a factor of 1.5 to 1.9 or ≥0.3 mg/dL within 48 h after the procedure	Technical success rate: CO_2_: 99/102 (97%) ICM: 201/211 (95.3%)	Severe procedure-related complications: CO_2_: 0/102 ICM: 0/211 Transient leg pain: CO_2_: 15/102 (14.7%) ICM: 0/211

Abbreviations: CLI, critical limb ischaemia; CO_2,_ carbon dioxide; cGy, centigray; GFR, glomerular filtration rate; ICM, iodinated contrast medium; IQR, interquartile range; NR, not reported; *n*, number of patients with events; *N*, number of patients at risk; mg, milligram; PAD, peripheral arterial disease; SD, standard deviation. ^a^: Percentages or number of events were self-calculated if not reported in the publication. ^b^: Timeframe not included in the definition, but creatinine was measured up to 1 month after the procedure. ^c^: One patient from the CO_2_ group was lost to follow-up. ^d^: Only patients with a serum creatinine lower than 2.3 mg/dL (200 µmol/L) were included. ^e^: Only patients with a serum creatinine of 1.7 mg/dL (150 µmol/L) or higher were included. ^f^: Creatinine was reported in µmol/L and converted to mg/dL. ^g^: Only patients with a serum creatinine of 1.5 mg/dL or higher were included in the CO_2_ group.
