# Supplementary material for: Risk of Contrast-Associated Acute Kidney Injury in Patients Undergoing Peripheral Angiography with Carbon Dioxide Compared to Iodine-Containing Contrast Agents: A Systematic Review and Meta-Analysis
Source: J Clin Med. 2022 Dec 4;11(23):7203. doi: 10.3390/jcm11237203 (PMC9738867; doi:10.3390/jcm11237203)
Supplement: Supplementary file 1 [file jcm-11-07203-s001.zip › jcm-2015371-supplementary.pdf]

Supplementary Material

Risk of Contrast-Associated Acute Kidney Injury in Patients Undergoing Peripheral Angiography with Carbon Dioxide Compared to Iodine-Containing Contrast Agents: A Systematic Review and Meta-Analysis

Gernot Wagner, Anna Glechner, Emma Persad, Irma Klerings, Gerald Gartlehner, Deddo Moertl, Sabine Steiner

Table S1. Search strategies	2–4
Table S2. Reasons for exclusion based on full-text assessment	5–6
Table S3. Inclusion and exclusion criteria as well as primary and secondary outcomes of included studies	7–8
Table S4. GRADE evidence profile and summary of findings for the comparison of CO_2 and conventional ICM for peripheral angiography with or without vascular intervention	9
Figure S1. Risk-of-bias assessment for RCTs	10
Figure S2. Risk-of-bias assessment for cohort studies	10
Figure S3. Forest plot of the sensitivity analysis including only low and moderate/some risk of bias studies	11
Figure S4. Forest plot of the sensitivity analysis with studies predominantly including patients with impaired renal function (GFR < 60 ml/min)	11
Figure S5. Forest plot of the sensitivity analysis with studies including more than 40% patients with diabetes	12
Figure S6. Forest plot with subgroups according to the type of intervention	12
Figure S7. Forest plot with subgroups according to the mean or median amount of iodinated contrast medium administered in the control group	13

Table S1. Search strategies

**Ovid MEDLINE(R) ALL 1946 to February 14, 2022**		
	#	**Searches**
A. CO2 angiography	1	exp Angiography/
	2	Carbon Dioxide/
	3	1 and 2
	4	((carbon dioxide or carbondioxide or CO2 or "CO(2)" or "CO 2") adj3 (arteriograph* or angiograph*)).ti,ab,kf.
	5	3 or 4
humans	6	limit 5 to "humans only (removes records about animals)"
language	7	(english or german).lg.
	8	6 and 7
SR-Filter	9	Systematic Review.pt.
	10	review.pt.
	11	(medline or medlars or embase or pubmed or cochrane or (scisearch or psychinfo or psycinfo) or (psychlit or psyclit) or cinahl or ((hand adj2 search$) or (manual$ adj2 search$)) or (electronic database$ or bibliographic database$ or computeri?ed database$ or online database$) or (pooling or pooled or mantel haenszel) or (peto or dersimonian or der simonian or fixed effect)).tw,sh. or (retraction of publication or retracted publication).pt.
	12	10 and 11
	13	meta-analysis.pt. or meta-analysis.sh. or (meta-analys$ or meta analys$ or metaanalys$).tw,sh. or (systematic$ adj5 review$).tw,sh. or (systematic$ adj5 overview$).tw,sh. or (quantitativ$ adj5 review$).tw,sh. or (quantitativ$ adj5 overview$).tw,sh. or (quantitativ$ adj5 synthesis$).tw,sh. or (methodologic$ adj5 review$).tw,sh. or (methodologic$ adj5 overview$).tw,sh. or (integrative research review$ or research integration).tw.
	14	9 or 12 or 13
SR-Results	15	8 and 14
RCT-Filter	16	exp randomized controlled trial/ or (random* or placebo).mp.
RCT-Results	17	8 and 16
cNRS-Filter	18	exp cohort studies/ or exp epidemiologic studies/ or exp clinical trial/ or exp evaluation studies as topic/ or exp statistics as topic/
	19	((control and (study or group*)) or (time and factors) or cohort or program or comparative stud* or evaluation studies or survey* or follow-up* or ci).mp.
	20	18 or 19
	21	(animals/ not humans/) or comment/ or editorial/ or exp review/ or meta analysis/ or consensus/ or exp guideline/ or hi.fs. or case report.mp.
	22	20 not 21
cNRS-Results	23	8 and 22
excluding case reports	24	case reports/ or (case? not control).ti,kf.
	25	8 not 24
Total	26	15 or 17 or 23 or 25

**Cochrane Library, February 15, 2022**	
ID	Search
#1	[mh Angiography] and [mh ^"Carbon Dioxide"]
#2	(("carbon dioxide" OR carbondioxide OR "CO2" OR "CO(2)" OR "CO 2") NEAR/3 (arteriograph* OR angiograph*)):ti,ab,kw
#3	#1 OR #2

**Embase.com, February 15, 2022**

No.	Query
#1	'angiography'/exp/mj AND 'carbon dioxide'/exp
#2	(('carbon dioxide' OR carbondioxide OR co2 OR 'co(2)' OR 'co 2') NEAR/12 (arteriograph* OR angiograph*)):ti,ab,kw
#3	('carbon dioxide':ti,ab,kw OR carbondioxide:ti,ab,kw OR co2:ti,ab,kw OR 'co(2)':ti,ab,kw OR 'co 2':ti,ab,kw) AND (arteriograph*:ti,ab,kw OR angiograph*:ti,ab,kw) AND contrast:ti,ab,kw
#4	#1 OR #2 OR #3
#5	'animal'/exp NOT 'human'/exp
#6	#4 NOT #5
#7	#6 NOT 'conference abstract'/it
#8	#7 AND ([english]/lim OR [german]/lim)
#9	'systematic review'/de OR 'meta analysis'/exp OR (((systematic OR 'state of the art' OR scoping OR literature OR umbrella) NEXT/1 (review* OR overview* OR assessment*)):ti,ab,kw) OR 'review* of reviews':ti,ab OR 'meta analy*':ti,ab,kw OR metaanaly*:ti,ab,kw OR (((systematic OR evidence) NEAR/1 assess*):ti,ab,kw) OR 'research evidence':ti,ab OR metasynthe*:ti,ab,kw OR 'meta synthe*':ti,ab,kw
#10	#8 AND #9
#11	'randomized controlled trial'/exp OR random*:ti,ab OR placebo*:ti,ab OR 'single blind*':ti,ab OR 'double blind*':ti,ab OR 'triple blind*':ti,ab
#12	#8 AND #11
#13	'cohort analysis'/exp OR 'controlled study'/exp OR 'comparative study'/exp OR 'clinical trial'/exp OR 'evaluation study'/exp
#14	control:ti,ab,kw AND (study:ti,ab,kw OR group*:ti,ab,kw) OR (time:ti,ab,kw AND factors:ti,ab,kw) OR cohort:ti,ab,kw OR program:ti,ab,kw OR 'comparative stud*':ti,ab,kw OR 'evaluation studies':ti,ab,kw OR survey*:ti,ab,kw OR 'follow up*':ti,ab,kw OR ci:ti,ab,kw
#15	#13 OR #14
#16	'editorial'/exp OR 'review'/exp OR 'meta analysis'/exp OR 'practice guideline'/exp OR 'case report'/exp OR 'case study'/exp
#17	#15 NOT #16
#18	#8 AND #17
#19	'case report'/exp OR 'case study'/exp OR (case$:ti,kw NOT control:ti,kw)
#20	#8 NOT #19
#21	#10 OR #12 OR #18 OR #20

**Epistemonikos, May 19, 2021**
Search
(("carbon dioxide" OR carbondioxide OR "CO2" OR "CO(2)" OR "CO 2") AND (arteriograph* OR angiograph*))
Filter: Systematic Review
Filter: Primary Study

**Pubmed Similar Articles (based on first 100 linked references for every seed article), May 18, 2021**	
Search number	Query
1	15640411
2	Similar articles for PMID: 15640411
3	33555574
4	Similar articles for PMID: 33555574
5	28463460
6	Similar articles for PMID: 28463460
7	26535013
8	Similar articles for PMID: 26535013
9	15640411 33555574 28463460 26535013 10207465 28463460 26946253 26567126 12148095 31843298 31583416 24704050 16949481 25380326 26535013 25449989 10584800 33556507 26567126 28463460 9840034 32064540 31843298 27380993 11793285 26567126 29636253 23246079 19463464 26431707 24704050 27318704 15713929 24076297 27821094 27590533 12650035 28939956 26112028 22504126 31712187 24704050 31054788 25380326 8548465 28550589 33555574 28550589 2596812 27995343 28939956 28479456 18256017 26667947 23324952 26460289 10693711 33527684 23994417 27671460 2354386 30641399 28550589 27692467 8944120 29636253 24076297 28735955 8624196 22504126 27995343 25589696 21288688 27318704 28231903 27986482

	8225887 28891160 31961262 24080134 21570871 31583416 25589696 28378609 9323247 26946253 27671460 10693711 9577501 30187381 29802583 25147039 26567126 25519207 29772311 27209402 10357491 29802583 27566903 28527930 11312200 25499712 22383347 31419029 1799190 29110930 30261736 26389534 15365517 32063447 28891160 28595481 10431273 31419029 28112470 24650745 8634409 30269828 31264950 25135875 12563205 22383347 30989349 27288105 9378023 30865912 24403826 25216216 16611682 26509137 31961246 22341837 15280530 31255335 29467030 20824749 2688531 30989328 25699983 24478190 17254739 33330951 26460289 12132028 17003814 26535013 29069419 29549415 25589696 24726211 20975263 8507115 9694446 31187227 26797158 30292616 8762947 28479456 23993293 24155171 14712411 32425437 25005762 17538131 21522098 31823699 28735955 26946253 8065882 9323247 25380326 28366300 12034949 33466210 26819427 27594393 9585778 27462884 32428641 23581754 11338095 15640411 27665399 23129039 11960230 25438909 30132106 27432201 28463460 21406544 26899537 25463341 12481147 32184976 25438909 27288107 10803187 30143056 18802377 27481496 2014280 31230933 22887964 28440113 12431148 29772311 30368506 24941300 10619301 32437415 30561410 24002391 23931876 10787201 25519207 29224939 10795832 30261736 24909428 27385151 23521455 26250726 15640411 28964617 14600187 25736516 22504126 27575814 17884368 27988086 20540038 25595408 8634406 23324952 28647690 22788900 12877615 32432003 21570871 31814551 1462006 23088805 32471671 9798858 7942829 29666905 26298226 25290790 12594698 26658089 23548372 15607391 6385671 28947366 27001054 27793622 15894775 24530668 33466210 21494517 10845486 33355750 23314316 23683380 8610396 30989349 23770181 22278664 7583725 28726528 25688117 26245919 9510571 24305547 28197679 20338130 1473919 30154014 27659469 24095039 8883529 29069419 23379629 27099285 8894938 33498733 25982920 26362632 7872367 29549415 30290766 31626944 15077867 30139467 27778420 22887964 20189850 10693711 29948863 29363894 12068157 27653990 21943940 20506547 8020522 29522871 31215488 27195520 11887978 31386574 24530668 27444365 16123870 26460289 22592896 17254739 7614794 31079840 25789733 26525064 8793433 30406762 28404621 26776895 1931218 21040324 23413087 26902938 7648588 26240732 27318704 22157477 12891113 17254739 23246079 28421855 31626944 27259368 32192658 27423995 9419625 26690912 28947366 29084493 11981070 25589696 23622917 26412436 21277800 31587663 25780349 26806239 8144334 32292766 33254372 25194550 19033249 12650035 23604313 19453527 2919599 29293980 25616679 27427347 12492082 28648650 30464400 28479446 11323513 22288088 32253719 26584581 8507115 31530501 32259900 25241319 12642275 33188865 31626944 28216361 8995472 33711514 31814551 25060042 8088970 30480977 27707552 28126175 23092640 33742201 29432373 25241317 11227644 33532287 23907993 21030201 11104872 33882497 22516682 33466210 2729314 33993231 28692484 32476034
10	#9 NOT ("Animals"[Mesh] NOT "Humans"[Mesh])
11	#10 AND ("english"[Language] OR "german"[Language])
12	#11 AND systematic[sb]
13	#11 AND (randomized controlled trial[Publication Type] OR (random*[Title/Abstract] AND controlled[Title/Abstract] AND trial[Title/Abstract]))
14	#11 AND (cohort[all] OR (control[all] AND study[all]) OR (control[tw] AND group*[tw]) OR epidemiologic studies[mh] OR program[tw] OR clinical trial[pt] OR comparative stud*[all] OR evaluation studies[all] OR statistics as topic[mh] OR survey*[tw] OR follow-up*[all] OR time factors[all] OR ci[tw]) NOT ((animals[mh:noexp] NOT humans[mh:noexp]) OR comment[pt] OR editorial[pt] OR review[pt] OR meta analysis[pt] OR case report[tw] OR consensus[mh] OR guideline[pt] OR history[sh])
15	#12 OR #13 OR #14

**ClinicalTrials.gov, February 15, 2022**
Search
("carbon dioxide" OR carbondioxide OR "CO2" OR "CO(2)" OR "CO 2") AND (arteriography OR angiography)

**WHO ICTRP** (https://trialsearch.who.int/), **February 15, 2022**
Search
carbon dioxide AND arteriograph* OR carbondioxide AND arteriograph* OR "CO2" AND arteriograph* OR "CO 2" AND arteriograph* OR "carbon dioxide" AND angiograph* OR carbondioxide AND angiograph* OR "CO2" AND angiograph* OR "CO 2" AND angiograph*

Table S2. Reasons for exclusion based on full-text assessment

Reference	Reason for exclusion
**Ineligible study design (9 studies)**	
Arendt C.T., Leithner D., Lenga L., Wichmann J.L., Albrecht M.H., Czwikla R., Varga-Szemes A., d'Angelo T., Martin S.S., Thalhammer A., Nagel E., Vogl T.J., and Gruber-Rouh T., Multi-observer comparison study between unenhanced quiescent-interval single-shot magnetic resonance angiography and invasive carbon dioxide angiography in patients with peripheral arterial disease and chronic renal insufficiency. European Journal of Radiology, 2018. 108: p. 140-146.	*Patients underwent both, quiescent-interval single-shot magnetic resonance angiography (QISS-MRA) and invasive CO_2 angiography.*
Burckenmeyer F., Schmidt A., Diamantis I., Lehmann T., Malouhi A., Franiel T., Zanow J., Teichgraber U.K.M., and Aschenbach R., Image quality and safety of automated carbon dioxide digital subtraction angiography in femoropopliteal lesions: Results from a randomized single-center study. European Journal of Radiology, 2021. 135: p. 109476.	*All patients received both, CO_2 and iodinated contrast medium.*
Ghumman S.S., Weinerman J., Khan A., Cheema M.S., Garcia M., Levin D., Suri R., and Prasad A., Contrast induced-acute kidney injury following peripheral angiography with carbon dioxide versus iodinated contrast media: A meta-analysis and systematic review of current literature. Catheter Cardiovasc Interv, 2017. 90(3): p. 437-448.	*Systematic review article.*
Huang S.G., Woo K., Moos J.M., Han S., Lew W.K., Chao A., Hamilton A., Ochoa C., Hood D.B., Rowe V.L., and Weaver F.A., A prospective study of carbon dioxide digital subtraction versus standard contrast arteriography in the detection of endoleaks in endovascular abdominal aortic aneurysm repairs. Annals of Vascular Surgery, 2013. 27(1): p. 38-44.	*All patients received both, CO_2 and iodinated contrast medium.*
Madhusudhan K.S., Sharma S., Srivastava D.N., Thulkar S., Mehta S.N., Prasad G., Seenu V., and Agarwal S., Comparison of intra-arterial digital subtraction angiography using carbon dioxide by 'home made' delivery system and conventional iodinated contrast media in the evaluation of peripheral arterial occlusive disease of the lower limbs. J Med Imaging Radiat Oncol, 2009. 53(1): p. 40-9.	*All patients received both, CO_2 and iodinated contrast medium.*
Oliva V., Common A., and Bettmann M.A., Prospective randomized crossover pilot study of the safety and efficacy of carbon dioxide versus iodinated contrast for peripheral angiography. Academic Radiology, 1998. 5 Suppl 1: p. S58-9; discussion S60-2.	*All patients received both, CO_2 and iodinated contrast medium.*
Oliva V.L., Denbow N., Therasse E., Common A.A., Harel C., Giroux M.F., and Soulez G., Digital subtraction angiography of the abdominal aorta and lower extremities: carbon dioxide versus iodinated contrast material. Journal of Vascular & Interventional Radiology, 1999. 10(6): p. 723-31.	*All patients received both, CO_2 and iodinated contrast medium.*
Rolland Y., Duvauferrier R., Lucas A., Gourlay C., Morcet N., Rambeau M., and Chaperon J., Lower limb angiography: a prospective study comparing carbon dioxide with iodinated contrast material in 30 patients. AJR. American Journal of Roentgenology, 1998. 171(2): p. 333-7.	*All patients received both, CO_2 and iodinated contrast medium.*
Scalise F., Commentary: Carbon Dioxide Automated Angiography in Patients With a High Risk of Contrast-Induced Nephropathy Who Undergo Percutaneous Interventions for Critical Limb Ischemia. Journal of Endovascular Therapy, 2016. 23(1): p. 49-51.	*Commentary article.*
**Ineligible comparison (5 studies)**	
Dowling K., Kan H., Siskin G., Stainken B., Ahn J., Herr A., and Clement Darling R., Safety of limited supplemental iodinated contrast administration in azotemic patients undergoing CO2 angiography. J Endovasc Ther, 2003. 10(2): p. 312-6.	*Comparison of CO_2 and CO_2 plus ICM.*

Reference	Reason for exclusion
Frankhouse J.H., Ryan M.G., Papanicolaou G., Yellin A.E., and Weaver F.A., Carbon dioxide/digital subtraction arteriography-assisted transluminal angioplasty. Annals of Vascular Surgery, 1995. 9(5): p. 448-52.	*Comparison of CO_2 and CO_2 plus ICM.*
Knipp B.S., Escobar G.A., English S., Upchurch G.R., Jr., and Criado E., Endovascular repair of ruptured aortic aneurysms using carbon dioxide contrast angiography. Annals of Vascular Surgery, 2010. 24(7): p. 845-50.	*Comparison of CO_2 and CO_2 plus ICM.*
Spinosa D.J., Matsumoto A.H., Angle J.F., Hagspiel K.D., McGraw J.K., and Ayers C., Renal insufficiency: usefulness of gadodiamide-enhanced renal angiography to supplement CO2-enhanced renal angiography for diagnosis and percutaneous treatment. Radiology, 1999. 210(3): p. 663-72.	*Gadodiamide was used as contrast medium. No iodinated contrast medium applied.*
Taha A.G., Saleh M., and Ali H., Safety and Efficacy of Hybrid Angiography in Chronic Lower Extremity Ischemia Patients at Risk of Contrast-Induced Acute Kidney Injury. Vascular & Endovascular Surgery, 2022. 56(2): p. 180-189.	*Comparison of CO_2 and CO_2 plus ICM.*
**Ineligible outcome (5 studies)**	
Bettmann M.A., D'Agostino R., Juravsky L.I., Jeffery R.F., Tottle A., and Goudey C.P., Carbon dioxide as an angiographic contrast agent. A prospective randomized trial. Investigative Radiology, 1994. 29 Suppl 2: p. S45-6.	*Risk of CA-AKI not reported.*
de Almeida Mendes C., de Arruda Martins A., Teivelis M.P., Kuzniec S., Nishinari K., Krutman M., Halpern H., and Wolosker N., Carbon dioxide is a cost-effective contrast medium to guide revascularization of TASC A and TASC B femoropopliteal occlusive disease. Ann Vasc Surg, 2014. 28(6): p. 1473-8.	*Risk of CA-AKI not reported.*
de Almeida Mendes C., de Arruda Martins A., Teivelis M.P., Kuzniec S., Varella A.Y., and Wolosker N., Carbon Dioxide as Contrast Medium to Guide Endovascular Aortic Aneurysm Repair. Ann Vasc Surg, 2017. 39: p. 67-73.	*Risk of CA-AKI not reported.*
Mendes Cde A., Martins Ade A., Teivelis M.P., Kuzniec S., Varella A.Y., Fioranelli A., and Wolosker N., Carbon dioxide contrast medium for endovascular treatment of ilio-femoral occlusive disease. Clinics (Sao Paulo, Brazil), 2015. 70(10): p. 675-9.	*Risk of CA-AKI not reported.*
Miller F.J., Mineau D.E., Koehler P.R., Nelson J.A., Luers P.D., Sherry R.A., Lawrence F.P., Anderson R.E., and Kruger R.A., Clinical intra-arterial digital subtraction imaging. Use of small volumes of iodinated contrast material or carbon dioxide. Radiology, 1983. 148(1): p. 273-8.	*Risk of CA-AKI not reported.*
**Ineligible language (1 study)**	
Krajina A., Lojík M., Raupach J., Chovanec V., and Mašková J., Lower extremity digital subtraction angiography with carbon dioxide in patients with renal insufficiency. Ceska Radiologie, 2001. 55(3): p. 167-173.	*The full-text article is written in Czech.*

Table S3. Inclusion and exclusion criteria as well as primary and secondary outcomes of included studies

Author, year, trial number	Recruitment period	Inclusion criteria	Exclusion criteria	Primary and secondary outcomes
Elboushi et al. 2021 [29]<br><br>NCT04458714	2015–2018	• Patients with aortoiliac arteries atherosclerotic disease (TASC A, B and C classified by computed tomography angiography, with good distal runoff<br>• Referred to endovascular aortoiliac revascularisation<br>• No history of allergy to contrast medium<br>• Normal kidney function	• TASC D aortoiliac lesions, requiring femoral endarterectomy<br>• Significant multilevel distal disease<br>• Severe chronic obstructive lung disease, chronic kidney failure, heart failure or pregnancy<br>• Patients younger than 18 years old	*Primary outcome:*<br>• Image quality<br>*Secondary outcomes:*<br>• Technical success rate<br>• Procedure-related complications (incidence of cardiac death, myocardial infarction, stroke, major amputation, nonocclusive mesenteric ischaemia, extended hospitalisation [>24 h] as a consequence of CO_2-guided treatment, hematomas, pseudoaneurysms, perforations, CA-AKI, target lesion revascularisation, and death within 3 months after the intervention)
Shafe et al. 2021 [31]<br><br>IRCT 20191107045359N1	NR	• Adult patients undergoing peripheral lower-limb angiography<br>• Glomerular filtration rate (GFR) of >30 ml/min/1.73 m^2 based on the MDRD equation	• Contrast exposure in the preceding 30 days<br>• Heart or kidney transplantation, proteinuria or cirrhosis<br>• Chronic haemodialysis<br>• Fluctuations in serum creatinine levels exceeding 15% in the preceding 2 days<br>• Intracardiac shunt<br>• Need for catheterisation higher than the renal arteries, including antegrade upper limb access (radial or brachial arteries).	*Primary outcome:*<br>• Risk of CA-AKI<br>*Secondary outcomes:*<br>• Death or the need for kidney replacement therapy during a 1-month follow-up period.<br>• Limb or abdominal pain due to CO_2 injection
Liss et al. 2005 [30]<br><br>NR	1999–2001	• Patients referred for renal angiography investigation for suspected renal artery stenosis	• Patients with serum creatinine levels > 200 µmol/l (2.3 mg/dl)<br>• Age younger than 15 years	• Risk of CA-AKI<br>• Serum creatinine level and creatinine clearance 1 day, 2 days, and 2–3 weeks after the intervention
Sterner et al. 2001 [36]<br><br>NR	1996	• Patients who underwent percutaneous endoluminal diagnosis and therapy	• Renal transplantation<br>• Any kind of dialysis<br>• Haemodialysis immediately after the endovascular procedure	• Risk of CA-AKI

Author, year, trial number	Recruitment period	Inclusion criteria	Exclusion criteria	Primary and secondary outcomes
		at Malmö University Hospital<br>• Serum creatinine level ≥ 150 µmol/l		
Chao et al. 2007 [32]<br><br>NR	2003–2005	• Patients treated by EVAR at the University of Southern California University Hospital	• NR	*Primary outcome:*<br>• Successful graft placement, renal function, and the need for graft revision<br>*Secondary outcomes:*<br>• Endoleak type and frequency<br>• Periinterventional morbidity and mortality
Stegemann et al. 2015 [35]<br><br>NR	2012–2013	• Patients who were admitted for endovascular treatment of lower extremity PAD from the Duesseldorf PTA Registry	• NR	• Technical success rate<br>• Total irradiation and intervention time<br>• Risk of CA-AKI<br>• Severe procedure-related complications
Diamantopoulus et al. 2020 [33]<br><br>NR	2012–2013	• Patients with symptomatic PAD undergoing endovascular interventions<br>• Impaired renal function (eGFR<60 ml/min/1.73 m²)	• Cases that had received intravenous iodinated contrast medium for other imaging investigations the week preceding the index angioplasty<br>• Patients under regular haemodialysis	*Primary outcome:*<br>• Risk of contrast medium–induced nephropathy<br>*Secondary outcomes:*<br>• Differences in serum creatinine values immediately and up to 30 days post-procedure<br>• Total volume of iodinated contrast medium<br>• Calculation of a safe cut-off value of contrast medium volume to be used in order to avoid CA-AKI development based on ROC curve analysis
Jakobi et al. 2021 [34]<br><br>NR	2015–2018	• Patients with symptomatic PAD of the lower extremities<br>• Referred for endovascular revascularisation<br>• CKD stage 3 or higher (eGFR < 60 ml/min/1.73 m²)	• NR	• Risk of CA-AKI

Abbreviations: CA-AKI, contrast-associated acute kidney injury; CO_2, carbon dioxide; CKD, chronic kidney disease; eGFR, estimated glomerular filtration rate; EVAR, endovascular aortic aneurysm repair; MDRD, Modification of Diet in Renal Disease; NR, not reported; PAD, peripheral arterial disease; PTA, percutaneous transluminal angioplasty; ROC, receiver operating characteristics; TASC, Trans-Atlantic Inter-Society Consensus

Table S4. GRADE evidence profile and summary of findings for the comparison of CO_2 and conventional ICM for peripheral angiography with or without vascular intervention

Certainty assessment							Summary of findings				
Participants (studies) Follow-up	Risk of bias	Inconsistency	Indirectness	Imprecision	Publication bias	Overall certainty of evidence	Study event rates (%)		Relative effect (95% CI)	Anticipated absolute effects	
							With CO_2	With ICM		Risk with ICM	Risk difference with CO_2
**Contrast-associated acute kidney injury**											
256 (3 RCTs [29–31]) Follow-up: up to 3 months	not serious	not serious	not serious	very serious[a]	none	⊕⊕◯◯ Low	5/122 (4.1%)	18/134 (13.4%)	**RR 0.33** (0.13 to 0.81)	13 per 100	**9 fewer per 100** (from 12 fewer to 3 fewer)
872 (5 cohort studies [32–36]) Follow-up: up to 6 months	serious[b]	not serious	not serious	serious[c]	none	⊕◯◯◯ Very low	27/251 (10.8%)	97/621 (15.6%)	**RR 0.78** (0.31 to 1.97)	16 per 100	**3 fewer per 100** (from 11 fewer to 15 more)

Abbreviations: CI, confidence interval; ICM, iodinated contrast medium; CO_2, carbon dioxide; GRADE, Grading of Recommendations Assessment, Development and Evaluation; RCT, randomised controlled trial; RR, risk ratio

a. Small sample size and few events resulting in a broad confidence interval
b. Three studies rated as critical risk of bias and two studies rated as moderate risk of bias
c. Broad confidence interval

Figure S1. Risk-of-bias assessment for RCTs [29–31].

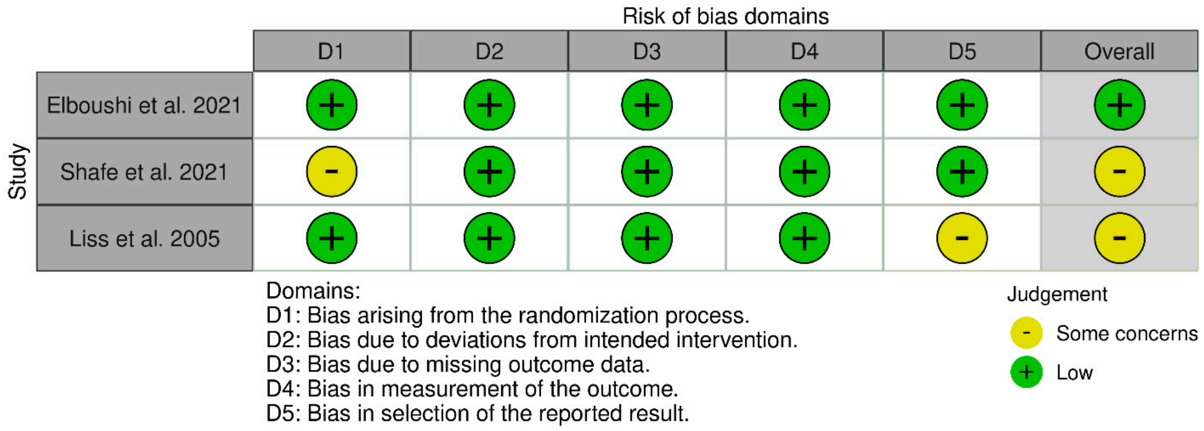

Abbreviations: RCT, randomised controlled trial

Figure S2. Risk-of-bias assessment for cohort studies [32–36].

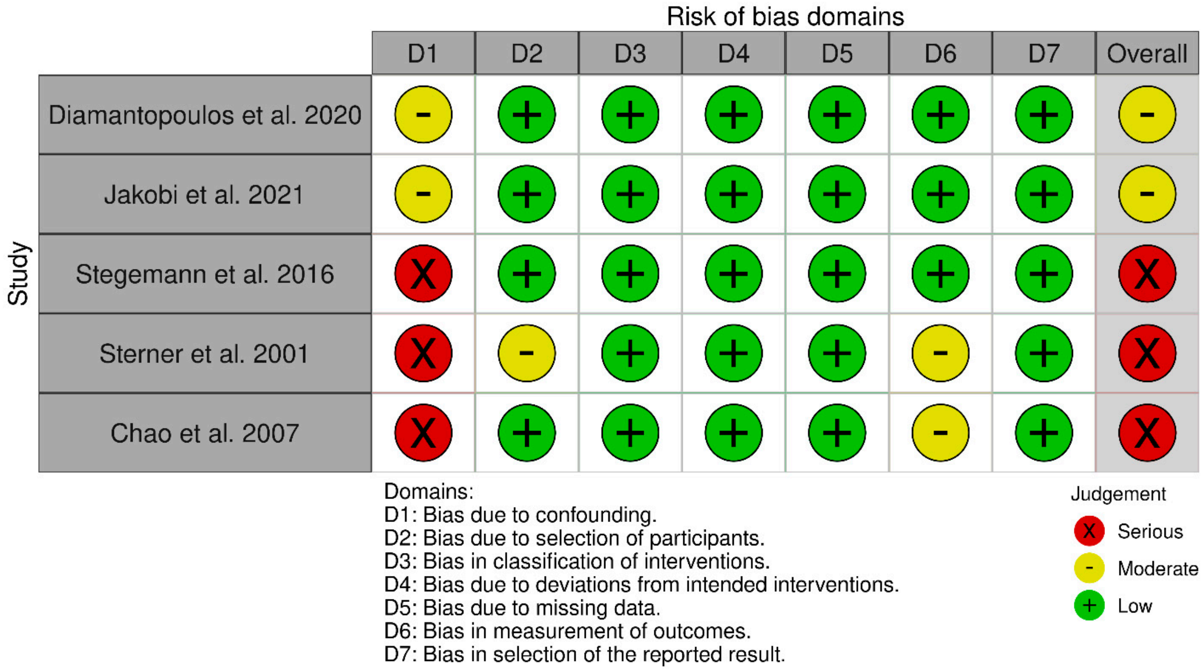

Figure S3. Forest plot of the sensitivity analysis including only low and moderate/some risk of bias studies [29–31,33,34].

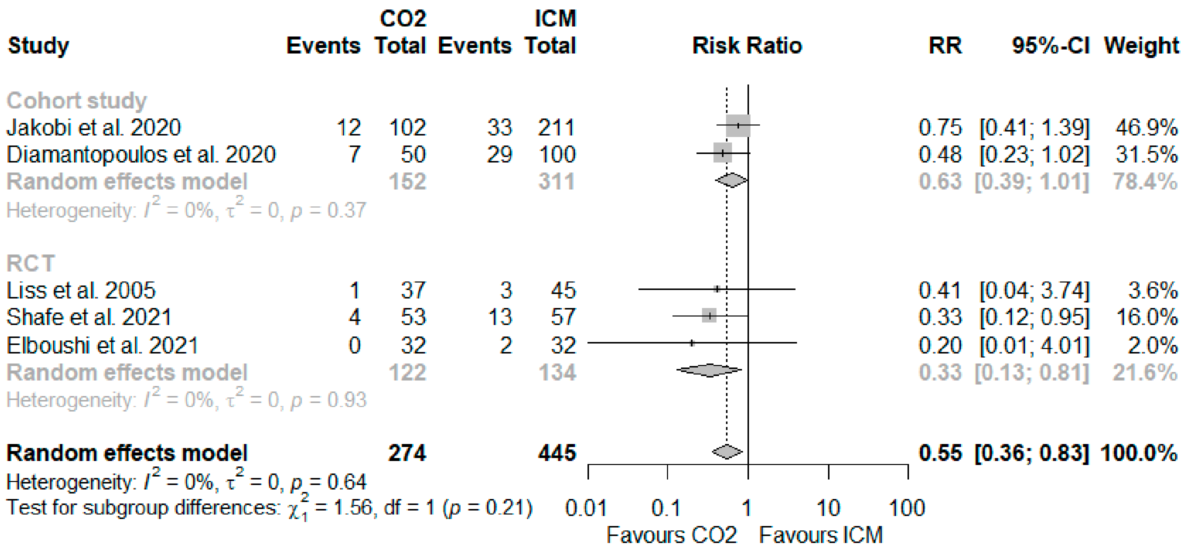

Abbreviations: CI, confidence interval; CO_2, carbon dioxide; ICM, iodinated contrast medium; RCT, randomised controlled trial; RR, risk ratio

Figure S4. Forest plot of the sensitivity analysis with studies predominantly including patients with impaired renal function (GFR < 60 ml/min) [30,33,34,36].

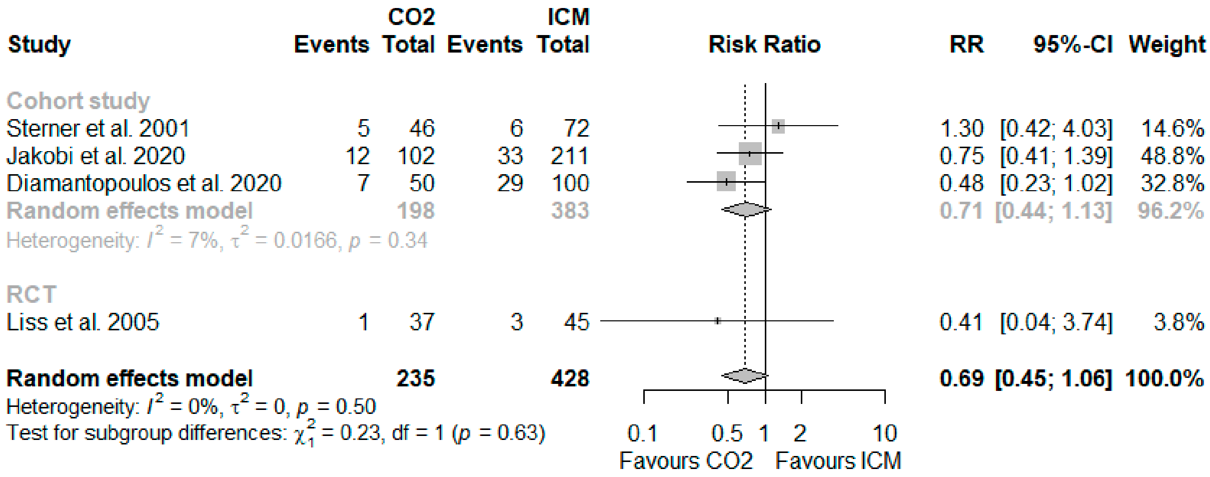

Abbreviations: CI, confidence interval; CO_2, carbon dioxide; GFR, glomerular filtration rate; ICM, iodinated contrast medium; RCT, randomised controlled trial; RR, risk ratio

Figure S5. Forest plot of the sensitivity analysis with studies including more than 40% patients with diabetes [29,31,33–35].

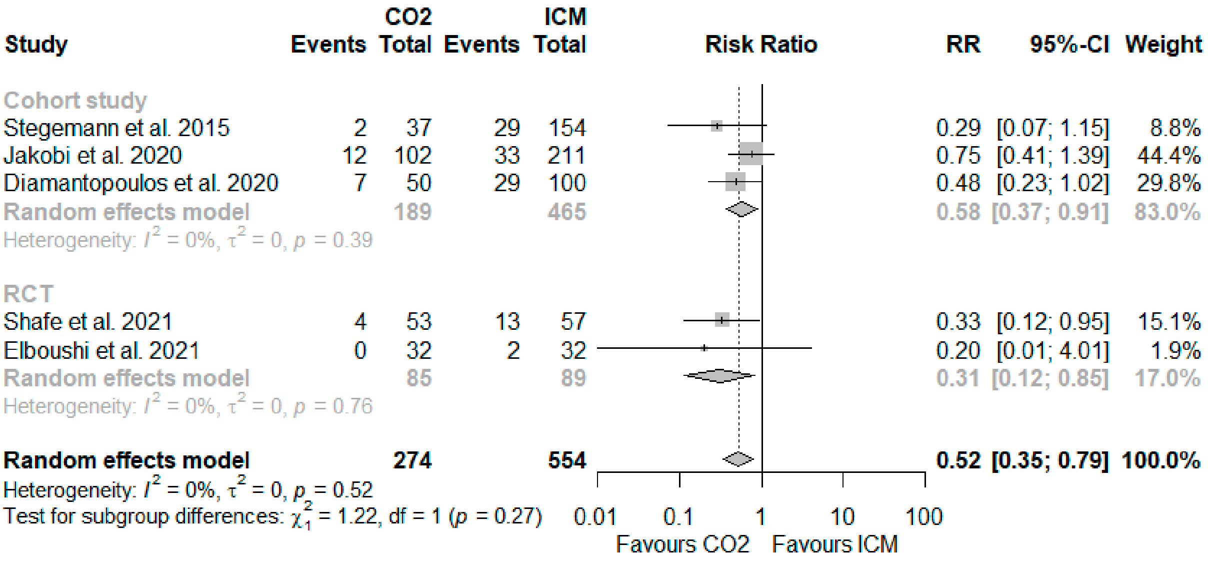

Abbreviations: CI, confidence interval; CO_2, carbon dioxide; ICM, iodinated contrast medium; RCT, randomised controlled trial; RR, risk ratio

Figure S6. Forest plot with subgroups according to the type of intervention [29–36].

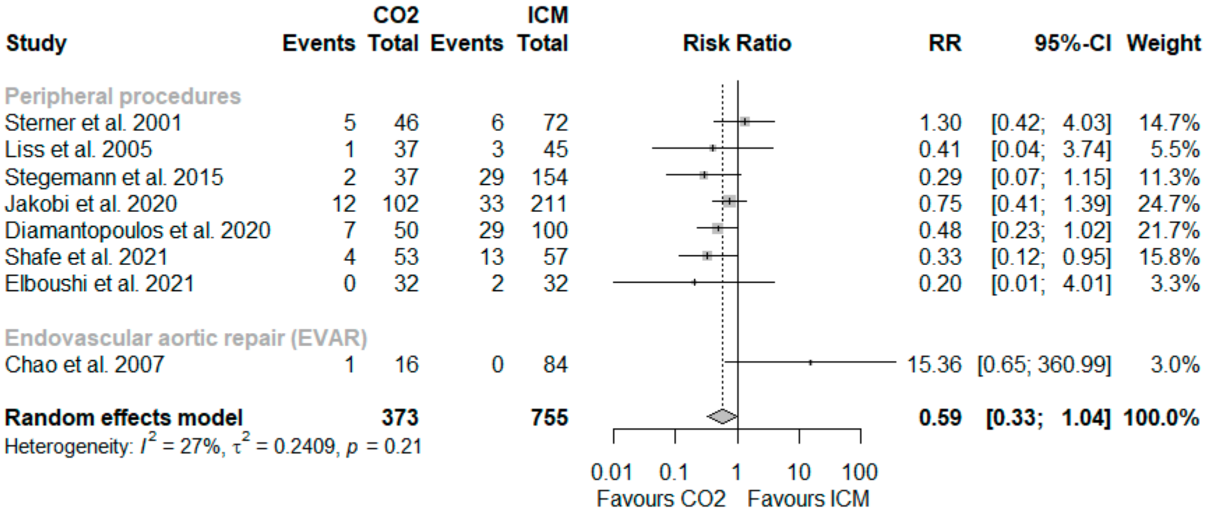

Abbreviations: CI, confidence interval; CO_2, carbon dioxide; ICM, iodinated contrast medium; RCT, randomised controlled trial; RR, risk ratio

Figure S7. Forest plot with subgroups according to the mean or median amount of iodinated contrast medium administered in the control group [29–36].

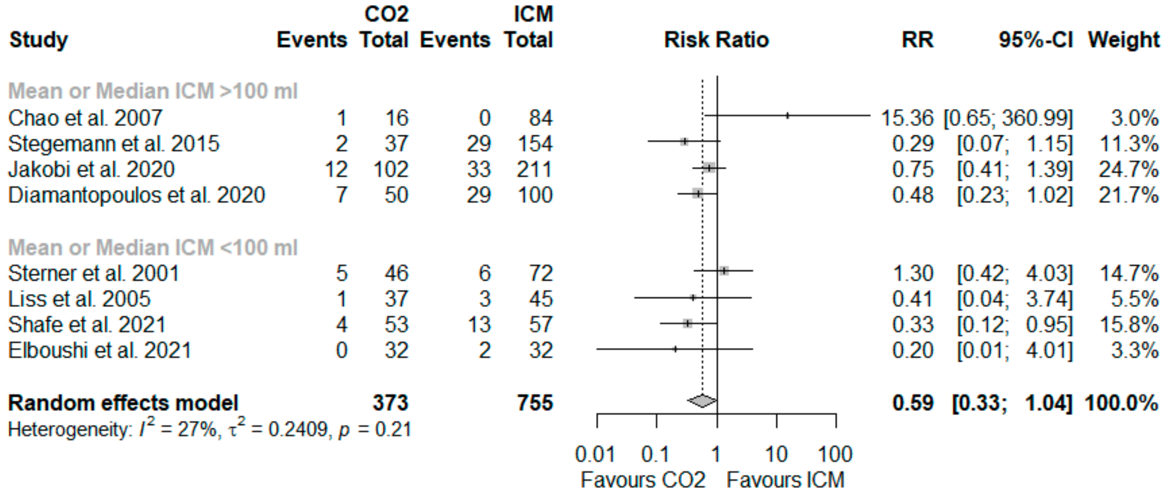

Abbreviations: CI, confidence interval; CO_2, carbon dioxide; ICM, iodinated contrast medium; RCT, randomised controlled trial; RR, risk ratio
